# Water column structure influences long-distance latitudinal migration patterns and habitat use of bumphead sunfish *Mola alexandrini* in the Pacific Ocean

**DOI:** 10.1038/s41598-021-01110-y

**Published:** 2021-11-09

**Authors:** Ching-Tsun Chang, Wei-Chuan Chiang, Michael K. Musyl, Brian N. Popp, Chi Hin Lam, Shian-Jhong Lin, Yuuki Y. Watanabe, Yuan-Hsing Ho, June-Ru Chen

**Affiliations:** 1 Department of Oceanography, University of Hawaii at Manoa, Honolulu, HI 96848 USA; 2 Eastern Marine Biology Research Center, Fisheries Research Institute, Chenggong Township, 22 Wuchuan Rd, Taitung, 961 Taiwan; 3 Pelagic Research Group LLC, PO Box 10243, Honolulu, HI 96816 USA; 4 Department of Earth Sciences, University of Hawaii at Manoa, Honolulu, HI 96848 USA; 5 Large Pelagics Research Center, PO Box 3188, Gloucester, MA 01931 USA; 6 National Institute of Polar Research, Tachikawa, Tokyo 190-8518 Japan; 7 Department of Polar Science, The Graduate University for Advanced Studies, SOKENDAI, Tachikawa, Tokyo 190-8518 Japan; 8 Fisheries Research Institute, 199 He 1st Rd., Keelung City, 202 Taiwan

**Keywords:** Behavioural ecology, Marine biology, Ichthyology

## Abstract

Satellite-tracking of adult bumphead sunfish, *Mola alexandrini*, revealed long-distance latitudinal migration patterns covering thousands of kilometers. Horizontal and vertical movements of four bumphead sunfish off Taiwan were recorded with pop-up satellite archival tags in 2019–2020. Two individuals moved northward and traveled to Okinawa Island and Kyushu, Japan and two moved southwards; crossing the equator, to Papua New Guinea and New Caledonia. During daytime, bumphead sunfish descended below the thermocline and ascended to mixed layer depths (MLD) during nighttime. The N–S migrants, however, demonstrated different habitat utilization patterns. Instead of using prevailing currents, the northward movements of sunfish cohorts exhibited extensive use of mesoscale eddies. Fish in anticyclonic eddies usually occupied deeper habitats whereas those in cyclonic eddies used near-surface habitats. On northward excursions, fish spent most of their time in regions with high dissolved oxygen concentrations. Southward movement patterns were associated with major currents and thermal stratification of the water column. In highly stratified regions, fish stayed below the thermocline and frequently ascended to MLD during daytime either to warm muscles or repay oxygen debts. These results for bumphead sunfish present important insights into different habitat use patterns and the ability to undergo long-distance migrations over varying spatial-temporal scales and features.

## Introduction

Bumphead sunfish *Mola alexandrini* are widely distributed throughout tropical and temperate regions. Several satellite tracking studies of sunfish species have been completed in the North Pacific and Atlantic Oceans^1–7^. Studies have shown that bumphead sunfish apparently prefer warmer waters compared to their congener (ocean sunfish, *M. mola*)^7,8^ but movement studies are limited^7,9,10^ (Table 1), and movement corridors and behaviors in the Pacific Ocean are not well characterized. Prior satellite tagging data indicated that bumphead sunfish traveled thousands of kilometers near the equatorial front and dove into mesopelagic depths (1112 m) in the Galapagos Islands^10^. A clear diel vertical movement pattern was described where tagged fish descended to the mesopelagic zone during daytime and ascended to the epipelagic zone during nighttime^9,10^.

**Table 1:** Summary tag information of *Mola alexandrini* from current and earlier studies*.*

Electronic device	ID	Body weight and length	Location	Tagging date	Tagging location	Pop off location	Pop off date	Duration (days)	Distance (km)/speed (km day^−1^)	References
PSAT	66588	450 kg, 220 cm	Taiwan	2019/4/2	24° 04′ N, 121° 37′ E	29° 20′ N, 130° 46′ E	2019/9/24	178	1079 km 6.06 km day^−1^	This study
PSAT	195549	290 kg, 180 cm	Taiwan	2019/12/16	22° 52′ N, 123° 09′ E	26° 35′ N, 127° 04′ E	2020/2/21	78	542 km 6.95 km day^−1^	This study
PSAT	195553	240 kg, 170 cm	Taiwan	2019/12/11	22° 50′ N, 122° 07′ E	00° 52′ S, 164° 35′ E	2020/5/9	150	5183 km 34.55 km day^−1^	This study
PSAT	195550	225 kg, 160 cm	Taiwan	2020/1/8	22° 35′ N, 122° 41′ E	21° 44′ S, 168° 16′ E	2020/9/4	240	6952 km 28.97 km day^−1^	This study
PSAT	45923	400–450 kg, > 200 cm	Taiwan	2018/3/28	24° 13′ N, 121° 46′ E	24° 06′ N, 121° 38′ E	2018/4/14	18	54 km 3 km day^−1^	Chang et al.^7^
Ultrasonic tag, GPS	31738–31741	98–154 cm	Galapagos Islands	2011/9/26	–	–	2011/10/1 2011/11/19	4 53	2740 km 51.7 km day^−1^	Thys et al.^10^
PSAT	52918–89298	100–150 cm	Indonesia	2004–2008	8° 42′ S–8° 42′ N, 115° 26′ E–115° 27′ E	8° 40′ S–10° 34′ S, 114° 17′ E-121° 25′ E	2004–2009	7–188	8.4–747 km 0.04–20.3 km day^−1^	Thys et al.^9^

Oceanographic characteristics including temperature, dissolved oxygen, thermal structure, eddies and prey availability drive the movement and distribution of many pelagic fishes^11,12^. Tracking studies of ocean sunfish indicated seasonal migration patterns to high latitudes in summer to locate preferred water temperatures and/or areas with high prey productivity^3,5^. The shift of temperatures in water column often constrains the vertical movement of fishes. The thermocline segregates warm water near the surface from deeper and cooler water, limiting oxygen transport and influencing thermal structure and gradients. Results of tagging studies showed that some predators have the ability to descend to great depth for exploiting prey organisms but need to return to the surface to warm muscles and/or to repay oxygen debts^13,14^ while other species are largely confined to MLD due to temperature and concomitant physiological limitations^15,16^. Furthermore, mesoscale eddies and frontal areas influence movement behaviors for many pelagic fishes and mammals^12,17,18^. Mixing processes of anticyclonic eddies and cyclonic eddies influence chlorophyll *a* concentration and create enhanced foraging opportunities for different sized predator and prey species in the oligotrophic open ocean^17^.

Correlating vertical movements to oceanographic characteristics provides insights into habitat utilization in the migration patterns for many pelagic fishes and sharks. Herein, we used pop-up satellite archival tags (PSATs) to study the horizontal and vertical movements of bumphead sunfish. Specifically, the influence of water temperature, thermal stratification, dissolved oxygen, and eddies on movement patterns and habitat uses of bumphead sunfish were investigated.

## Results

### Horizontal movements

**Figure 1:**
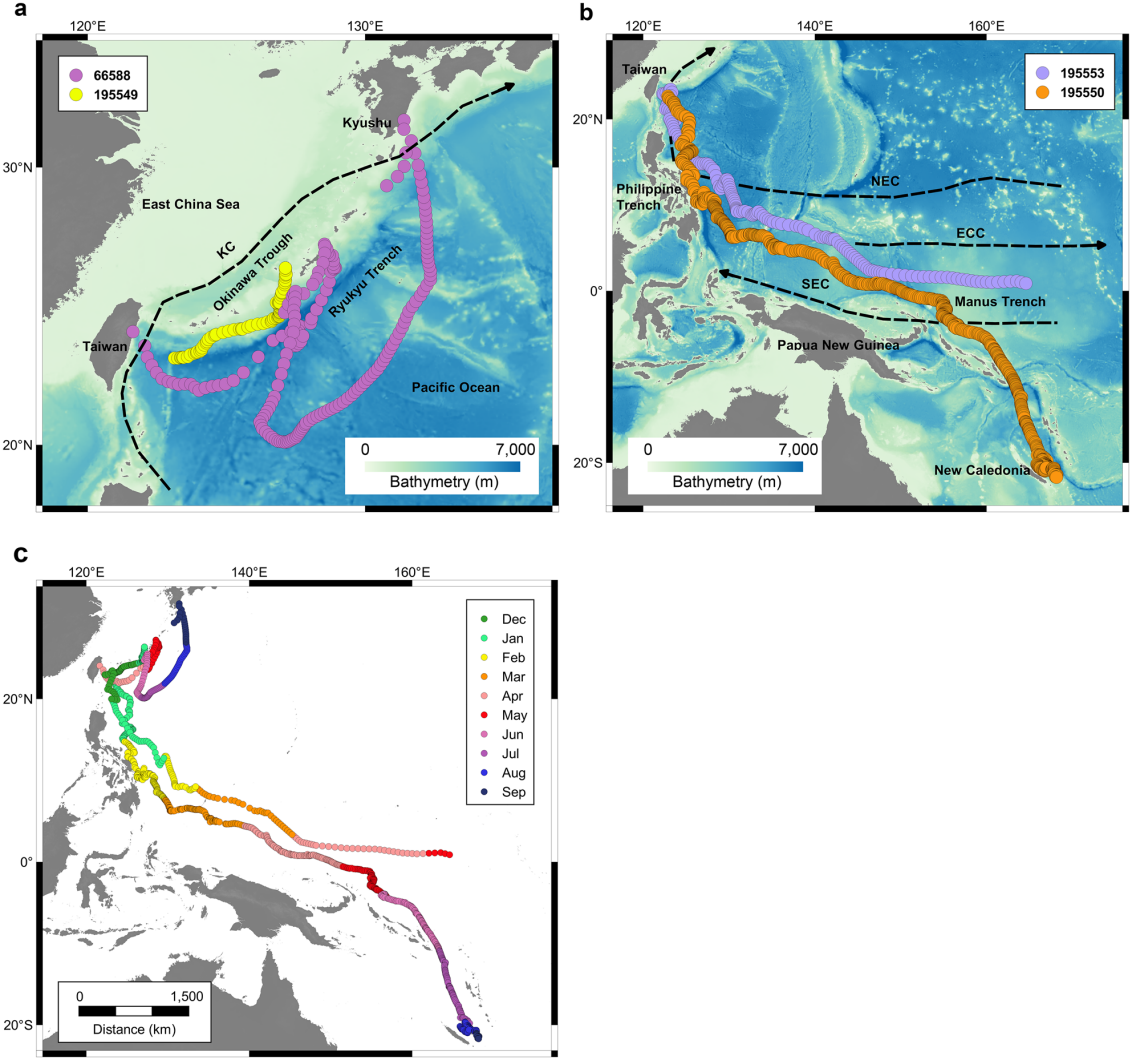
Most probable tracks of *Mola alexandrini*. (**a**) Tracks of fish 66588 and fish 195549 with bathymetry. (**b**) Tracks of fish 195550 and fish 195553 with bathymetry. (**c**) Track of all individuals color-coded by months. KC: Kuroshio Current. NEC: North Equatorial Current. ECC: Equatorial Counter Current. SEC: South Equatorial Current. Figure was created with QGIS 2.18.0. 2016. Quantum GIS Geographic Information System. Open Source Geospatial Foundation Project. http://www.qgis.org/en/site/.

From 2019 to 2020, four PSATs were deployed on bumphead sunfish ranging from 160 to 220 cm total length (Table 1) and PSATs stayed attached for 78–240 days at-liberty. Fish 66588 and 195549 moved northwards from the tagging location and traveled straight-line distances of 1079 km and 542 km with average speeds of 6 and 7 km day^−1^, respectively (Fig. 1a). Fish 66588, the largest individual (Table 1), moved to Okinawa Island, Japan in May, and then to Kyushu Island, Japan in August and September (Fig. 1c). Fish 195549 moved northwards to Okinawa Island in January (Fig. 1a). Both individuals experienced ambient water temperatures ranging from 7 to 30 °C. The migratory pathway appeared to follow the Ryukyu Trench.

Two smaller individuals, fish 195550 and 195553 traveled 6952 km and 5183 km from tagging locations to Papua New Guinea and New Caledonia with speeds of 29 and 35 km day^−1^, respectively. These individuals moved southward to the east of the Philippines in January and February, and then moved closer to the equator in April and May, and finally to New Caledonia in August and September (Fig. 1b, c). These fish experienced water temperatures ranging from 5 to 31 °C.

### Vertical habitat

**Figure 2:**
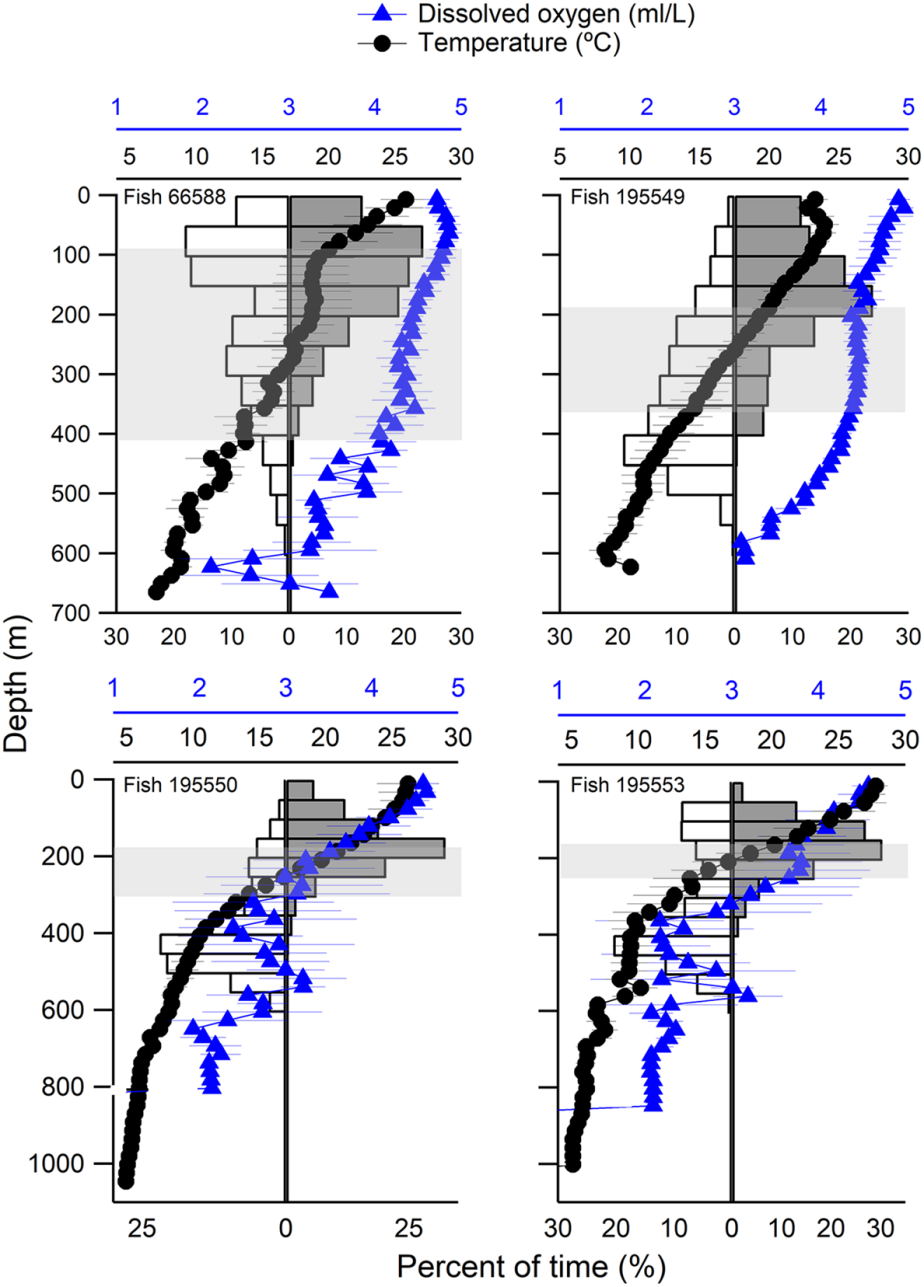
Depth profile of *Mola alexandrini* in daytime (white bar) and nighttime (dark gray bar) with ambient water temperature (circle) and dissolved oxygen (triangle). Error bars in profile indicate standard deviations in temperature and dissolved oxygen. Shaded areas show approximate temperature at thermocline top (20 °C) and bottom (14 °C).

Tagged individuals exhibited diel vertical movement patterns (Table 2). All individuals dove deeper in daytime than nighttime (Kruskal–Wallis: *p *< 0.05) where individuals stayed mainly in and/or above the thermocline during nighttime and below the thermocline during daytime (Fig. 2). Individuals (fish 66588 & 195549) that moved northward spent more time near the surface (15–20%) compared to individuals that moved southward (1–3%, fish 195550 & 195553). Average sea surface temperatures (SST) visited by fish moving northward (fish 66588: 24 ± 3 °C SD; fish 195549: 23 ± 1 °C) were significantly cooler than fish moving southward (fish 195550: 26 ± 2 °C; fish 195553: 28 ± 1 °C) (*p* < 0.05).

**Table 2:** Mean day depth, night depth, depth range, and temperature range of *Mola alexandrini.*

Fish	Day depth (m)	Night depth (m)	Depth range (m)	Temperature range (˚C)	References
66588	212.2 ± 144.2	145.2 ± 89.3	3–670	6.8–29.5 (18.7 ± 4.1)	This study
195549	328 ± 118.7	170.7 ± 99	0.5–623	7.1–25.7 (17.9 ± 4.4)	This study
195550	385.3 ± 119.5	169.1 ± 74.8	4–1100	4.9–30.8 (16.9 ± 6.3)	This study
195553	328.6 ± 142.1	167.6 ± 71	1–1052	5.1–30 (18.5 ± 4.7)	This study
45923	232.5 ± 106.9	93.2 ± 63.5	0–486	7.7–34.1 (16.2 ± 4.2)	Chang et al.^7^
31739	–	–	− 1112	4.5–23.2	Thys et al.^10^
52918, 52943	–	–	0–450	10–27.5	Thys et al.^7^

Fish 66588 spent most of its time within/above the thermocline, where water temperature ranged from 15 to 23 °C in both daytime and nighttime. Fish 195549 spent about 73% of time above 200 m during nighttime in comparison to 20% during daytime. Fish 195550 and fish 195553 both spent 40–58% of their time below 400 m during daytime with temperatures < 10 °C and 85% of time > 250 m during nighttime (15–28 °C).

### Environmental variables influencing vertical activity

Results from the GAMM models (Figs. S1, S2) indicated the relationships between depth and environmental variables. The best fit models were selected based on Akaike information criterion (AICc) and Akaike weights (Table S1). The maximum depths visited were significantly influenced by thermocline, MLD, dissolved oxygen concentration (DO), and SST (best-fit model, adjusted R^2^ = 32%). Dissolved oxygen had a negative effect and the thermocline had positive effect on the maximum depth visited by tagged individuals (Fig. S1a). For tagged individuals that moved northwards, daytime movements (adjusted R^2^ = 79%) were significantly correlated with DO, sea level anomalies (SLA), MLD, thermocline depth, and SST. Nighttime movements were mainly influenced by thermocline, MLD, and SLA (adjusted R^2^ = 37%). Both DO and MLD had negative effects on the mean daytime and mean nighttime depth (Fig. S1b, c). Response plots showed a dome-shaped relationship between depth and SLA. For tagged individuals that moved southwards, daytime depths were related to DO, SLA, thermocline, and SST (adjusted R^2^ = 36%). Nighttime depth (adjusted R^2^ = 54%) was highly related with DO, MLD, thermocline, and SST. Thermocline was positively correlated with the depth of tagged individuals (Fig. S1d, e).

### Movement behavior and oceanography

**Figure 3:**
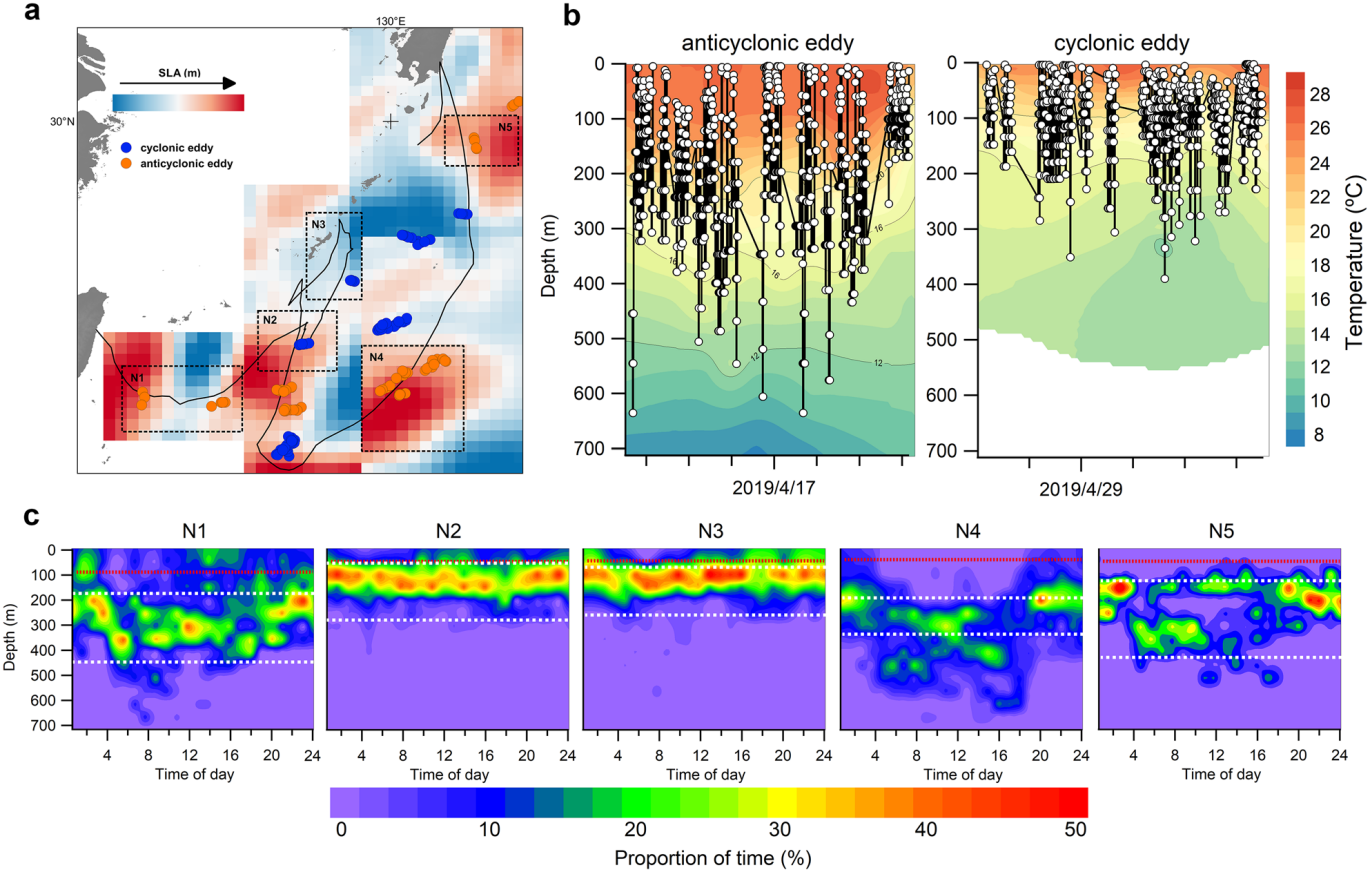
Time-at-depth distribution of fish 66588 in different time periods. N1: 4/8–20; N2: 4/25–30; N3: 5/27–31; N4: 8/1–25; N5: 9/11–15. (**a**) The most probable track of fish 66588 and the presence of anticyclonic eddies (red circle) and cyclonic eddies (blue circle). (**b**) Vertical movements and the water temperature in depth profiles of anticyclonic- and cyclonic eddies. (**c**) The time-at-depth distribution in different time periods. White dash-line represents the depth range of thermocline and red dash-line represents the depth of mixed layer depth.

**Figure 4:**
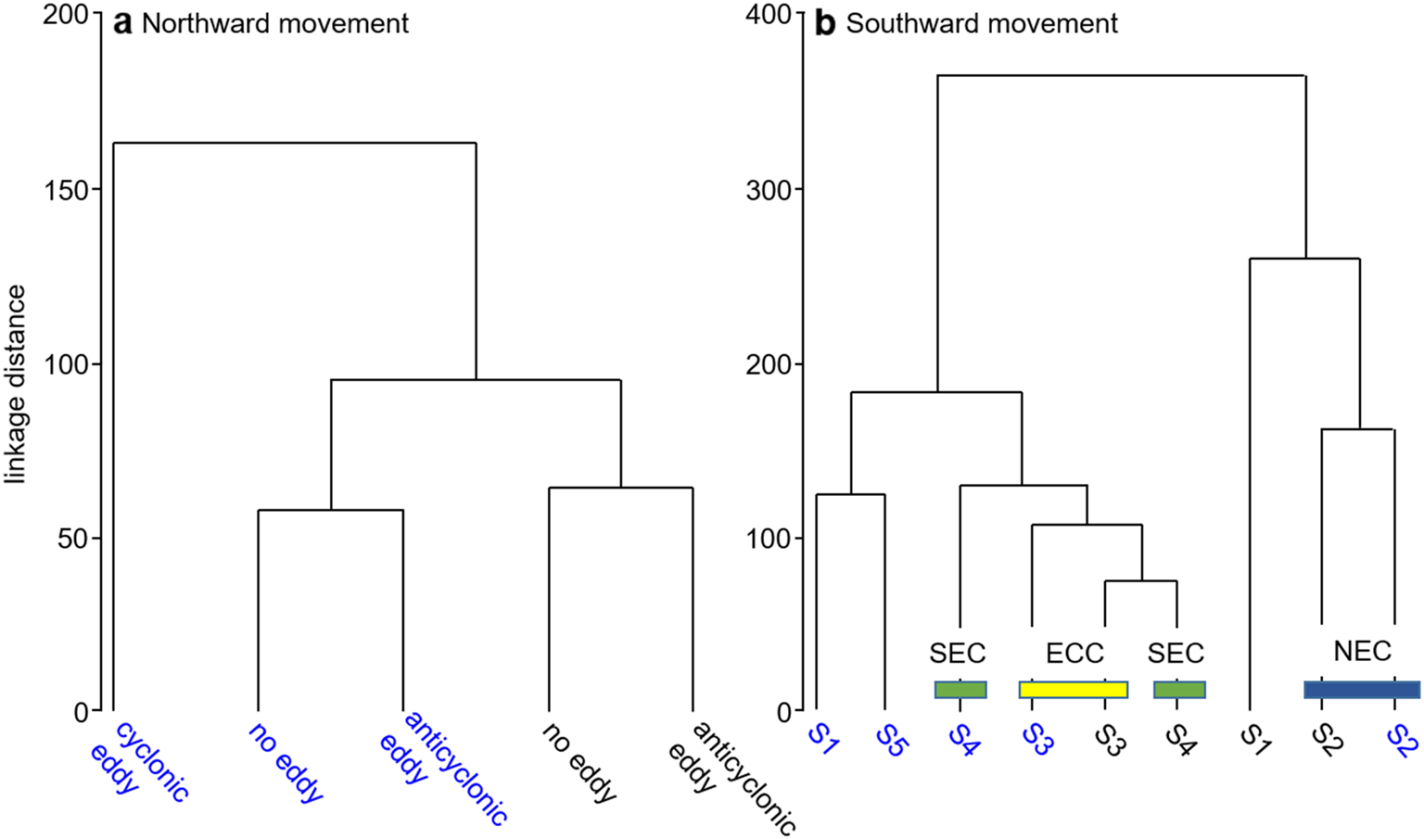
Cluster analysis dendrogram of movement behavior patterns for *Mola alexandrini* in different periods. (**a**) The northward movement behavior patterns of fish 66588 (blue) and fish 195549 (black) in anticyclonic eddies, cyclonic eddies, and absence of eddies (cophenetic correlation = 0.83). (**b**) The southward movement behavior patterns of fish 195550 (blue) and fish 195553 (black) in different current regions (cophenetic correlation = 0.82). NEC: North Equatorial Current. ECC: Equatorial Counter Current. SEC: South Equatorial Current.

Tagged bumphead sunfish showed distinct movement patterns related to oceanographic characteristics. During northward movements, tagged individuals did not travel with the Kuroshio Current for an assist but instead extensively used the periphery of mesoscale eddies (Fig. 3a). In sunfish-associated eddies (Table S2), MLD and thermocline were shallower in cyclonic eddies (cold-core) than anticyclonic eddies (warm-core). Fish 66855 spent 6% and 9% of its time in cyclonic and anticyclonic eddies, respectively. Fish 195549 spent 6% of its time in only anticyclonic eddies. Both individuals showed different vertical movements when associated with anticyclonic and cyclonic eddies (Fig. 4a). Fish 66588 showed different depth distributions in cyclonic and anticyclonic eddies during daytime and nighttime (Kolmogorov–Smirnov: *p* < 0.05). When occupying cyclonic eddies, fish 66588 made frequent vertical movements and spent more time (> 40%) at the surface to 200 m in daytime and nighttime with water temperature ranging from 17 to 23 °C (Fig. 3b, c). In the proximity of anticyclonic eddies, tagged individuals spent less time near the surface and went to greater depths (~ 600 m) and experienced water temperatures ranging from 10 to 32 °C.

**Figure 5:**
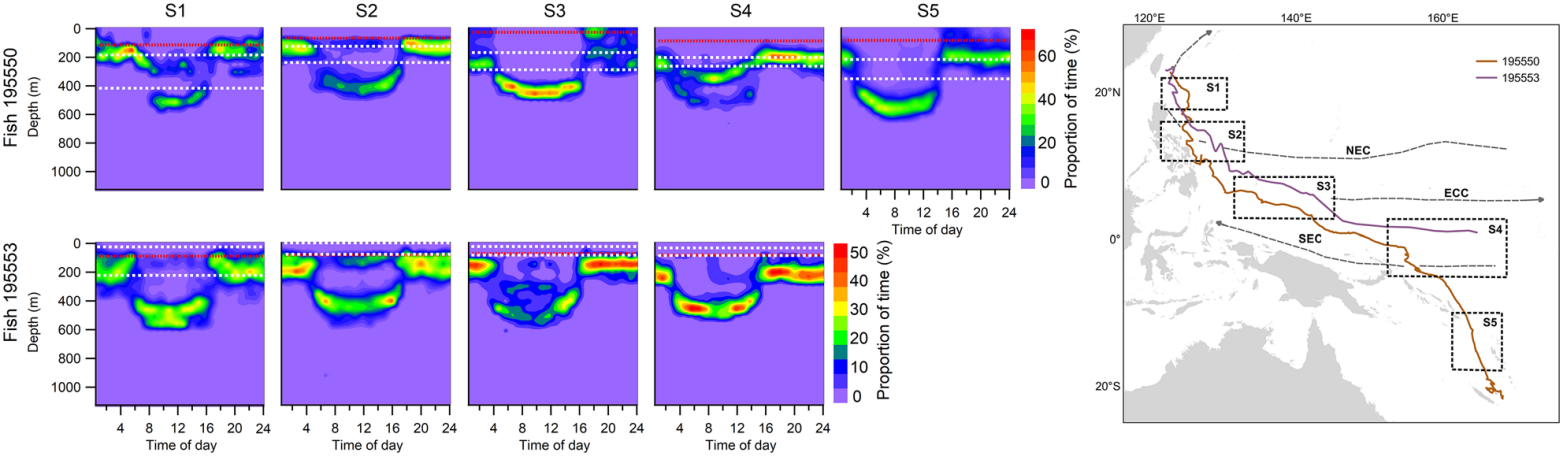
Time-at-depth distribution of tag 195550 and tag 195553 in different time periods. White dash-line represents the depth range of thermocline and red dash-line represents the depth of mixed layer. S1 (fish 195550: 1/11–14; fish 195553: 12/18–1/16) represents the tagged individuals that moved southward. S2 (fish 195550: 2/10–25; fish 195553: 1/26–2/25) represents the tagged individuals that swam cross the North Equatorial Current. S3 (fish 195550: 3/4–18; fish 195553: 3/15–4/3) represents the tagged individuals that moved along the Equatorial Counter Current. S4 (fish 195550: 5/8–20; fish 195553: 4/11–4/30) represents the tagged individuals that moved cross the South Equatorial Current. S5 (fish 195550: 7/1–31) represents the tagged individuals that moved to southern hemisphere. NEC is North Equatorial Current. ECC is Equatorial Counter Current. SEC is South Equatorial Current. Orange line in the right bottom plot represents the most probable track of fish 195550 and purple line represents the most probable track of fish 195553.

**Figure 6:**
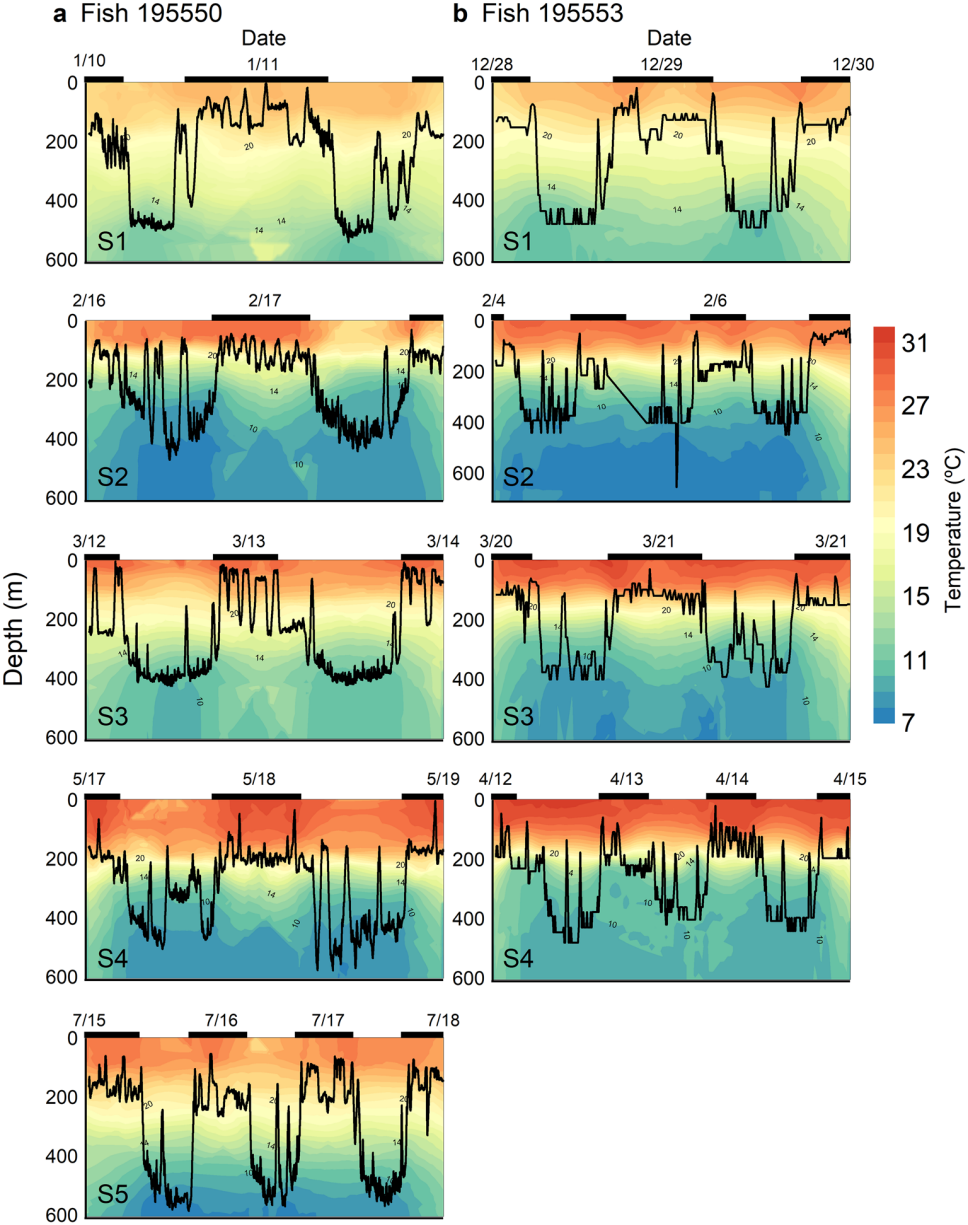
Vertical movement of fish 195550 (**a**) and fish 195553 (**b**) in different time periods with water temperature.

By contrast, the southwest movements of fish 195550 and 195553 were not eddy-associated, but instead appeared to be influenced by ocean currents. Both individuals moved southward in the North Equatorial Current (NEC) in February (S2) and traveled a southwest course in the Equatorial Counter Current (ECC) in March (S3), and then moved southward in the South Equatorial Current (SEC) in May (S4) (Fig. 5). Tagged individuals mainly stayed below MLD. In S1 and S5 (the periods not influenced by major currents), MLD and thermocline were deeper than when associated with currents (Fig. 5). Tagged individuals spent most time below 400 m (bottom of thermocline) with water temperatures from 10 to 15 °C during daytime and occupied the 150–250 m strata (18–25 °C) during nighttime. Moving from the NEC to the ECC, and finally to the SEC, thermal stratification increased (average MLD increased from 33 to 94 m). Tagged individuals mainly stayed at 100–200 m during nighttime and shifted to 350–400 m with water temperatures < 10 °C during daytime. During both daytime and nighttime, tagged individuals made frequent vertical forays within the MLD (Fig. 6). In the ECC, fish 195550 spent 9% of its time near the surface (0–50 m) at nighttime with a shallow MLD (33 m). The depth and temperature distribution patterns in SEC and ECC regions were similar (Fig. 4b).

### Dissolved oxygen

Dissolved oxygen concentration was related to water column structure. DO was the highest in the MLD and decreased with depth (Fig. 2). For tagged individuals that moved northward, they experienced DO ranging from 2 to 5 ml L^−1^ and they spent most time (> 90%) above 400 m and with DO of 4–5 ml L^−1^. Fish 195550 and 195553, which moved southward, experienced DO of 1–5 ml L^−1^. They spent 35–46% of their time in higher DO environments (4–5 ml L^−1^) during the night and spent 26–33% in 2 ml L^−1^ DO environments during the day.

## Discussion

This study documented the first long-distance latitudinal movement patterns of bumphead sunfish. Two bumphead sunfish moved against the prevailing current to the southern hemisphere and traveled 6952 km and 5183 km to New Caledonia and Papua New Guinea, respectively, and undertook the longest migration recorded for Molidae. Despite being released a month apart, they showed temporal synchronicity, and took similar movement paths along the coast of the Philippines and crossed the equator in April and May. Thys et al.^10^ recorded one bumphead sunfish travelled 2700 km to the equatorial front and suggested equatorial upwelling might provide suitable foraging areas. Two larger individuals in our study (fish 66588 and 195549) moved northward and exhibited eddy-associated behavior which to our knowledge is the first observation of the species utilizing mesoscale eddies. Our findings suggest that understanding movement patterns and effects of oceanographic characteristics requires detailed investigation along latitudinal routes to adequately characterize both the vertical and horizontal habitat requirements to predict movements.

Two individuals (fish 66588 and 195549) moved to high latitudes in summer paralleling the direction and course of the Kuroshio Current but they tended to avoid the current. Both individuals migrated to Okinawa Island, consistent with fisheries catch records^19,20^ and previous tagging studies^7^. Tagged individuals also moved to Kyushu Island, Japan in summer where similar routes were reported for skipjack tuna in terms of thermal preference and food availability^21^. The high productivity and plankton biomass in the Kuroshio Current, the continental self, and its neighboring water mass have been reported^22,23^, suggesting a preferred corridor with sufficient food availability for pelagic fishes in the northwestern Pacific Ocean. Several studies on *M. mola*^3,5,6^, blue shark^24^, and leatherback sea turtle^25^ documented meridional movements during summer where movements to higher latitudes might be related to thermal preferences and food availability. Another movement interpretation suggests northward migration and the September occurrence in Japanese waters might be driven by reproduction. Mature female sunfish were found in summer (July to October) off Japan^8,26^, implying an important spawning ground. Comparable spawning migration patterns have been observed in Pacific bluefin tuna^27,28^.

When fish 66588 moved northward, it spent 20% of its time near the surface within a narrow range of ambient water temperatures (23–24 °C). Bumphead sunfish displayed similar preferences in water temperature from 14 to 24 °C in Taiwan^7^ and from 20 to 25 °C off Indonesia^9^. By contrast, *M. mola* spent most time near the surface with temperature from 12 to 17 °C in the northeast Atlantic^5^, from 15 to 17 °C in Japan^3^, and from 12 to 20 °C in Taiwan^7^. Based on fisheries reports in Japan, bumphead sunfish mainly occurred in waters from 16 to 26 °C and *M. mola* occurred in water from 15 to 21 °C^8^. Together these findings suggest that bumphead sunfish may prefer higher temperatures than *M. mola*.

Sunfish generally stayed below the thermocline during the daytime and in/or above the thermocline during nighttime. Blue shark, bigeye tuna, and swordfish also demonstrated similar vertical movement patterns that exploited resources in the thermocline during daytime^15,29–31^. These movements probably mirrored those of vertically migrating organisms of the deep scattering layer for enhanced foraging opportunities.

Bumphead sunfish exhibited different vertical movement patterns and habitat utilization between southward and northward migrations. During northward movements, fish mainly stayed above 200 m in daytime and nighttime and were associated with mesoscale eddies. Pelagic species such as sharks, anchovy, salmon, mackerel, skipjack tuna, and sea turtles utilize eddies and perform distinct movement behaviors in anticyclonic and cyclonic eddies^17,18,32^. Anticyclonic eddies enhance foraging opportunities compared to cyclonic eddies due to the warmer temperatures and high prey densities^33,18^. Eddies are also influenced by the expansion and contraction of thermal structures which might affect the habitat preference and movement behavior of sunfish^12^.

Unlike northward migrations, bumphead sunfish that moved southwards did not display distinct vertical movements in or near eddy features. Instead, fish exploited geostrophic currents where depth distributions in the NEC, ECC, and SEC were shallower than other regions. Similar movement patterns were also found in sea turtles^34^. Bumphead sunfish often made deep excursions to the thermocline and ascended to MLD in highly stratified waters. The behavior can be regarded as a trade-off between physiological limitations and energy demands. Sunfish dove deep experiencing lower temperature and oxygen during daytime for foraging excursions and ascended to the surface to warm muscles and/or to repay oxygen debts. Many oceanic predators have similar strategies where they maintain warm body temperatures near the surface and exploit food resources at deeper depths^35^. For *M. mola*^13^, the thermal environment for foraging (5–12 °C) was lower than the average body temperature (16–17 °C). Whole-body heat-transfer coefficients were larger during the warming process than the cooling process. The large body size and surface area and thick skin might reduce the loss of heat, allowing fish to increase foraging time in cold water. Similar physiological mechanisms were found in sharks^36^. The cycles of deep foraging excursions and surface rewarming is possibly related to optimal foraging behavior.

Bumphead sunfish spent more time near the surface in the ECC areas characterized by wind-induced upwelling and shallow thermocline structure. Upwelling brings cold, nutrient-rich water to the surface attracting larger prey organisms, possibly allowing sunfish to feed at shallower depths.

The limitation of DO on the vertical movements and distribution of pelagic fishes has been demonstrated where oxygen minimum zones constrained vertical movements and habitat use^4,37,38^. It has been reported that many pelagic fishes tend to show avoidance responses to hypoxia zones with minimum oxygen concentrations < 2.5 ml L^–1^^39^. Although bumphead sunfish displayed a wide range in vertical movements, they preferred to stay in regions with high DO concentration (4–5 ml L^–1^) which is similar to other epipelagic predators, e.g., dolphinfish and billfish^40–42^. During northward migrations, bumphead sunfish spent most of their time in high DO environments where water temperature and mixing of eddies influenced DO concentrations^43,44^. Bumphead sunfish experienced a wide range of DO concentration from 2 to 5 ml L^−1^ and during southward migration, fish remained in low DO depth during the daytime. Like *M. mola*, they probably experienced lower DO concentrations as they moved nearshore^4^. The results suggested that sunfish can tolerate low DO concentrations on an ephemeral basis for feeding or to escape predators, also a pattern observed in striped marlin^40^.

## Conclusion

Satellite studies of bumphead sunfish demonstrated N–S migration patterns in the Pacific Ocean. Generally, vertical habitat use was related to the depth of the thermocline and MLD. Tagged fish stayed beneath the thermocline during daytime and above the thermocline or close to MLD during nighttime. Bumphead sunfish exhibited different habitat utilization patterns between southward and northward migrations. The northward movement behaviors showed an affinity to mesoscale eddies and thermal preferences while the southward movements were possibly related to the ocean currents and thermal stratification of the water column.

## Materials and methods

### Satellite tagging

Four tags were deployed on bumphead sunfish by harpoon and longline fisheries from September 2019 to January 2020 off Taiwan (Table 1). Pop-up satellite archival tags (PSATs; miniPAT, Wildlife Computer, Redmond, WA, USA) were programmed to release after 150–240 days. PSATs were tethered with 300-lb test monofilament and stainless steel darts^7^. Before tagging, the darts were treated with a broad-spectrum antibiotic to prevent infection and the tag was affixed beneath the dorsal fin. Round body weight was estimated by the captain and the body length was estimated from weight-length relationships (Chang, unpublished data). Pressure (converted to depth) and temperature were recorded every 10 min, and the MLD temperature and profiles of depth and temperature were summarized every 12 h. Two tags were physically recovered and provided archival data every 3 s (632,840 records) for fish 195550 and every 5 s (6,948,813 records) for fish 195549.

### Geolocation estimates

After pop-off, PSATs transmitted archived data via Argos, including times of sunrise and sunset, pressure (depth), temperature, light proxy, and MLD. Geolocations were estimated using Wildlife Computers GPE3 cloud-based software (Wildlife Computers 2019) on the transmitted data from detached tags, or from the full archival data record from recovered tags. The GPE3 software uses a proprietary state-space, hidden Markov model that includes ambient light, sea surface temperature, bathymetry, and Argos location to estimate most probable tracks. Location estimates were refined using the species’ swimming speeds^45^. The most probable tracks were created with QGIS^46^.

### Environmental parameters

To better understand the movement patterns and relationships of environmental variables, we used SLA (global, 0.25° resolution), geostrophic currents, SST (global, 0.01° resolution), and topography from the NOAA CoastWatch ERDDAP server (http://coastwatch.pfeg.noaa.gov/erddap). DO was estimated from World Ocean Atlas 2013 (WOA, global, 1° resolution). The DO data provided monthly means in 5-m bins for 0–100 m, in 25-m bins for 100–500 m, and then 50-m bins for 500–1150 m. Mesoscale eddies were identified from SLA^12,47^ and data from the Ocean Data Bank of the Ministry of Science and Technology, Republic of China (http://www.odb.ntu.edu.tw/). The depth of the MLD and thermocline in the water column were used to characterize and investigate the vertical movement behavior of tagged fish. The depth ranges between 20 °C isotherm and 14 °C isotherm was used to represent the approximate depth range of thermocline^48^.

## Data analysis

Day and night differences in temperature and depth distributions were compared using non-parametric Kruskall–Wallis tests and non-parametric two-sample Kolmogorov–Smirnov tests^49^. The time-at-depth and time-at-temperature datasets were calculated from time series data. Diel periods were split by the time of local sunset and sunrise by the NOAA Solar Calculator (https://gml.noaa.gov/grad/solcalc/). Diel depth and temperature were plotted as frequency histograms. Depth and temperature distributions associated with anticyclonic/cyclonic eddies and different current regions were calculated. To quantify and classify movement behavior and water column use during different periods; depth and temperature distributions, maximum and minimum depth/temperature, MLD, and thermocline were used to generate similarity trees with Euclidean distances for hierarchical cluster analysis^50^.

Generalized additive mixed models (GAMMs, R package, mgcv and MuMIn) were used to determine potential factors that influenced vertical movements. Three GAMMs were fit separately to daily maximum depth, daytime mean depth and nighttime mean depth and tagged individuals were set as random effects. To understand the effects of water column structure on vertical movement, five environmental variables were set as fixed effects including SLA, SST, DO at 100 m depth, MLD, and thermocline. Each variable was included in a stepwise manner and the AICc, delta AIC, and Akaike weights were calculated. The final GAMM model was selected by lowest AICc and highest Akaike weights. We used F tests to determine whether the models were well fitted^51^.

## Ethics statement

The protocols used in this study conform to the legal requirements and institutional guidelines of the Fisheries Research Institute, Council of Agriculture (COA), Taiwan. The protocols have been reviewed and approved by the Institutional Animal Care and Use Committee of Fisheries Research Institute, COA. All procedures involving animals were carried out in accordance with relevant guidelines and regulations (including ARRIVE guidelines).

## Supplementary Information

Supplementary Information.
