# Supplementary material for: Water column structure influences long-distance latitudinal migration patterns and habitat use of bumphead sunfish Mola alexandrini in the Pacific Ocean
Source: Sci Rep. 2021 Nov 9;11:21934. doi: 10.1038/s41598-021-01110-y (PMC8578541; doi:10.1038/s41598-021-01110-y)
Supplement: Supplementary file 1 — Supplementary Information. [file 41598_2021_1110_MOESM1_ESM.pdf]

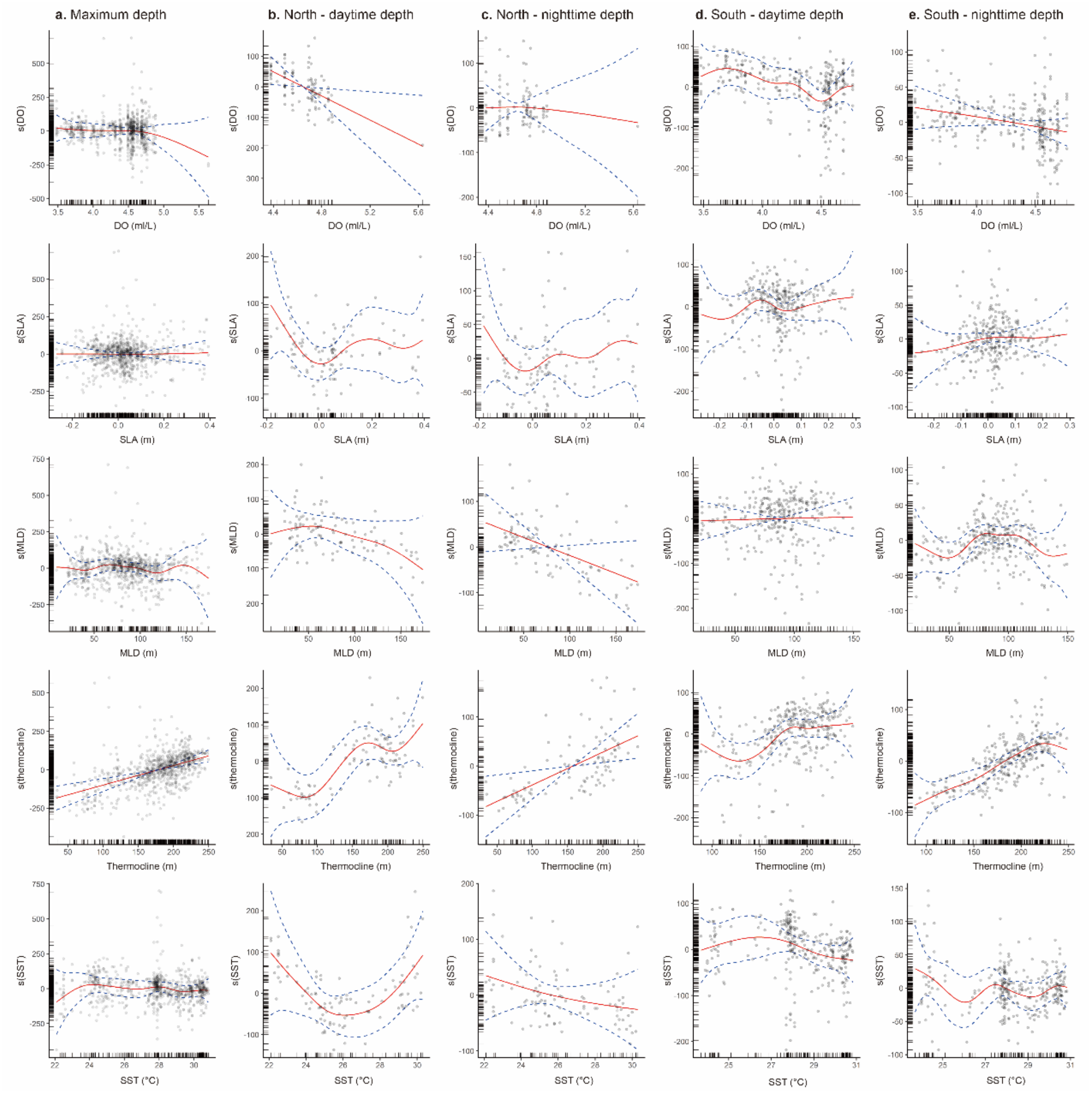

Supplementary Figure S1. Generalized additive mixed models plots showing the effects of environmental variables on daily maximum depth, daytime depth and nighttime depth of *Mola alexandrini*. Five plots are available for each model (from up to down): (1) DO: dissolved oxygen, (2) SLA: sea surface anomalies, (3) MLD: mixed layer depth, (4) thermocline, and (5) SST: sea surface temperature. Red solid lines show the predicted values from fitted models and dashed blue lines 95% confidence intervals.

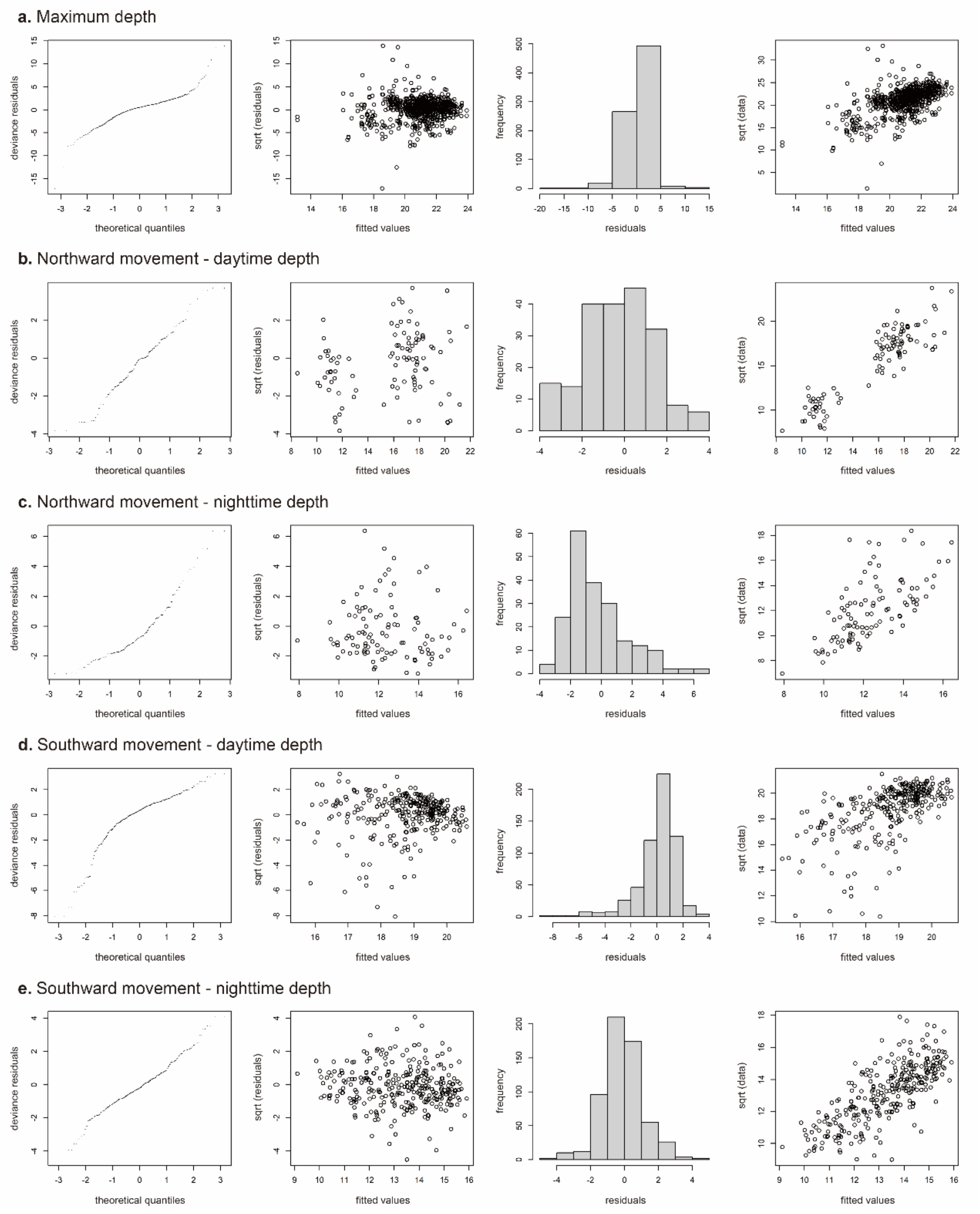

Supplementary Figure S2. Diagnostic plots for GAMM models on daily vertical activity (maximum depth, daytime depth, nighttime depth) of *Mola alexandrini*. Four plots are available for each model (from left to right): (1) Q-Q plot for deviance residuals of the full model, (2) fitted values versus square-root-transformed residuals, (3) distribution of residuals, (4) fitted values versus square-root-transformed observed values.

Supplementary Table S1 Relative importance of each environmental variable to the final model, including Akaike information criterion (AICc), delta AIC (ΔAIC), and Akaike weights (wAIC).

	AIC	ΔAIC	wAIC
**Maximum depth**			
Full	9467.9	0	0.56
No DO	9475.5	7.62	0.01
No SLA	9468.5	0.65	0.41
No MLD	9478.7	10.88	0.00
No thermocline	9585.9	118.06	0.00
No SST	9474.5	6.59	0.02
**Northward movement - Mean daytime depth**			
Full	2173.7	0.00	1.00
No DO	2204.0	30.24	0.00
No SLA	2195.8	22.05	0.00
No MLD	2185.4	11.70	0.00
No thermocline	2213.4	39.72	0.00
No SST	2220.2	46.43	0.00
**Northward movement - Mean nighttime depth**			
Full	2128.3	0.00	0.58
No DO	2129.7	1.32	0.30
No SLA	2132.8	4.46	0.06
No MLD	2141.8	13.43	0.00
No thermocline	2166.9	38.56	0.00
No SST	2133.1	4.76	0.06
**Southward movement - Mean daytime depth**			
Full	6452.1	0.00	0.55
No DO	6486.8	34.72	0.00
No SLA	6460.8	8.70	0.00
No MLD	6452.4	0.39	0.45
No thermocline	6512.6	60.50	0.00
No SST	6465.5	13.42	0.00
**Southward movement - Mean nighttime depth**			
Full	5804.0	0.00	0.94
No DO	5815.6	11.64	0.00
No SLA	5809.7	5.71	0.06
No MLD	5843.9	39.87	0.00
No thermocline	5986.8	182.79	0.00
No SST	5817.7	13.69	0.00

Supplementary Table S2 Records of anticyclonic- and cyclonic eddies of fish 66588 and fish 195549 occurred. Center SSHa represents sea surface height anomaly of the eddy center.

Fish	Type	Radius (km)	Amplitude (cm)	Center SSHa (m)
66588	anticyclonic	101.7	16.5	0.45
66588	cyclonic	48.6	6.4	-0.05
66588	cyclonic	83.8	21.3	-0.2
66588	anticyclonic	100.9	6.4	0.3
66588	cyclonic	82.6	7.3	-0.15
66588	anticyclonic	126.2	16	0.4
66588	cyclonic	70.7	14.6	-0.15
66588	cyclonic	127.6	20.1	-0.1
66588	anticyclonic	236.1	27.1	0.5
66588	cyclonic	69.2	24.4	-0.5
195549	anticyclonic	191.2	6.9	0.2
